# Contractures and Deformities of the Hand

**Published:** 1909-06-19

**Authors:** 


### Surgery.

### CONTRACTURES AND DEFORMITIES OF THE HAND.

The hand is liable to a large number of conditions which impair its usefulness; and these, though many of them are slight in themselves, may have serious results in preventing the patient from following his occupation successfully, or at any rate may impair his wage-earning capacity.

The best known of these deformities is undoubtedly Dupuytren's contracture. This consists of a flexion of the fourth and fifth fingers, often of both hands. It is not always bilateral, however; but when it is so, it starts in one hand before the other. At first the little finger becomes gradually contracted down into the palm, and sooner or later the ring finger follows suit. Extension is impossible, and in an advanced case the tip of the finger can hardly be raised from the palm. The disease starts most often in early adult life, and little is known of its causation and pathology, except that in many cases a hereditary element can be discovered in the shape of a family history; in other cases gout seems to be a determining factor. Occasionally this contracture comes on in later life, and then it appears to be definitely due to chronic irritation of the palm such as is produced by the use of a walking stick or by the patient's occupation; for instance, the pressure of the handle of an instrument, as in carpenters.

When an attempt is made to straighten the deformed fingers, it will be found that the inner half of the palmar fascia stands out as a thickened band, for it is the palmar fascia and not the flexor tendons that is the primary cause of the trouble. It is easy enough to get the fingers straight again by operation, but it is essential that this should be radical. It is not sufficient to tenotomise the constricting bands subcutaneously. An open operation must be performed. The actual incision is not a matter of great importance, provided that a satisfactory exposure of the parts is obtained. An incision parallel to the axis of the limb over the most prominent portion of the thickened fascia, with two short ones at right angles to the ends of the first incision, so that two skin-flaps can be turned back, will meet the demands of the case very well. The whole of the thickened fascia must then be carefully dissected away. When this is done it will generally be found that the fingers can be straightened out completely. So far little trouble is experienced. But it is in *keeping* the fingers straight and in preventing a recurrence of the deformity that the great difficulty is experienced.

The hand should be put up on a splint which keeps the fingers extended; and this should be kept on until the wound is healed. It may then be taken off, but the fingers must be actively extended several times daily for some months. But the really important point in the treatment is to prevent contraction of the fingers during sleep. The tonicity of the flexors is much greater than that of the extensors, and when voluntary control is removed by sleep the hand tends naturally to contract. A simple but effective contrivance to overcome this may be made as follows: A flat piece of wood is cut out to the shape of the hand. This is strapped to the back of the hand each night. On its anterior surface five leather ringlets about one and a-half inches in length are applied. They are so placed that when the fingers are inserted into them their centre corresponds to the line of the terminal interphalangeal joint. This will effectually prevent the fingers from bending. This apparatus must be worn every night until the fingers remain straight without its support. The length of time necessary will vary in each case, but it is rarely less than six months. A good result should never be promised, because the operation itself causes a certain amount of scar tissue in the palm, and this tends in time to contract and reproduce the original deformity.

Most of the other deformities met with in the hand are the direct result of septic infection or of injury. It is the rule rather than the exception to find some deformity or loss of function in a finger which has been the site of a whitlow, especially if the infection has invaded the sheath of the flexor tendon. In such a case the severity of the inflammation prevents the adoption of early passive movement. When the inflammatory process has subsided it is often found that the tendon is adherent to the anterior surface of one or more of the phalanges, and immobility results. In these cases an anæsthetic should be given and the adhesions should be broken down. Afterwards passive movement and massage should be persisted in to prevent their re-formation; but a perfect result, as far as function is concerned, is rarely obtained.

Ischæmic contracture of the flexors of the forearm sometimes occurs after simple fractures. In most cases it results from the injudicious use of splints; the usual cause being that splints are applied and not taken down often enough. For it must be remembered that a fracture is followed by swelling of the limb. Splints may be applied immediately after the fracture, and they may then not be too tight. But if the limb swells up afterwards the splint may and does cause injurious pressure on the muscles, depriving them of their nutrition and leading to atrophy and contraction. In all cases of fracture, therefore, the splints should be taken off on the next day to examine the condition of the limb. But ischæmic contracture occasionally occurs when no splints have been applied. This is sometimes seen after fractures of the forearm in young infants, especially after separation of the lower epiphysis of the radius. The inflammatory reaction is so great that the muscles of the forearm are damaged and rendered atrophic.

However produced, a contraction of the flexors of the forearm and wrist follows, which is very intractable. The muscles are so much shortened that even under an anæsthetic extension is impossible. The only treatment is to perform a tenoplasty, but the result is nearly always unsatisfactory.

